# Sex differences in addiction-relevant behavioral outcomes in rodents following early life stress

**DOI:** 10.1016/j.addicn.2023.100067

**Published:** 2023-01-26

**Authors:** Millie Rincón-Cortés

**Affiliations:** Department of Neuroscience, School of Behavioral Brain Sciences, University of Texas at Dallas, 800 W Campbell Road BSB 14, Richardson, TX 75080, United States of America

**Keywords:** Early life stress, Maternal separation, Limited bedding, Reward, Addiction, Sex differences

## Abstract

In humans, exposure to early life stress (ELS) is an established risk factor for the development of substance use disorders (SUDs) during later life. Similarly, rodents exposed to ELS involving disrupted mother-infant interactions, such as maternal separation (MS) or adverse caregiving due to scarcity-adversity induced by limited bedding and nesting (LBN) conditions, also exhibit long-term alterations in alcohol and drug consumption. In both humans and rodents, there is a range of addiction-related behaviors that are associated with drug use and even predictive of subsequent SUDs. In rodents, these include increased anxiety-like behavior, impulsivity, and novelty-seeking, altered alcohol and drug intake patterns, as well as disrupted reward-related processes involving consummatory and social behaviors. Importantly, the expression of these behaviors often varies throughout the lifespan. Moreover, preclinical studies suggest that sex differences play a role in how exposure to ELS impacts reward and addiction-related phenotypes as well as underlying brain reward circuitry. Here, addiction-relevant behavioral outcomes and mesolimbic dopamine (DA) dysfunction resulting from ELS in the form of MS and LBN are discussed with a focus on age- and sex-dependent effects. Overall, these findings suggest that ELS may increase susceptibility for later life drug use and SUDs by interfering with the normal maturation of reward-related brain and behavioral function.

## 1. Introduction

In humans, exposure to early life stress (ELS) is linked to greater susceptibility for developing a variety of psychiatric disorders, which often emerge during adolescence and persist into adulthood [1–5]. For example, ELS is a potent predictor of later life alcohol and/or drug dependence and addiction in humans [6,7]. In adults exposed to adverse childhood experiences (ACEs), ACE scores are strongly related to initiation of drug use from early adolescence into adulthood and problems with drug use [8–12]. Compared to individuals without a history of ELS, people with 5 or more ACEs were up to 10 times more likely to initiate illicit drug use and become addicted to drugs [8]. Similar effects have also been found for alcohol use: ACEs increased the likelihood of initiating alcohol use during early life (age 14) and adult alcohol dependence [13,14]. The neurobiological basis for these effects is unknown but is thought to involve aberrant function of the hypothalamic-pituitary-adrenal (HPA) axis, which mediates the stress response, and the mesolimbic dopamine (DA) system, which mediates reward-related processes including neurobehavioral responses to drugs of abuse [6,7,15–17].

In humans, longitudinal studies have indicated that the first postnatal years of life constitute a sensitive period for the enduring effects of ELS experienced within the context of the caregiver such as parental absence, maltreatment, neglect, and physical abuse, among others [18,19]. Many of these stressors can be modeled in developing rodents to provide mechanistic insights into disrupted behavioral and brain processes by which ELS may contribute to later life substance use disorders (SUDs). For example, maternal separation (MS) and limited bedding and nesting (LBN) are two widely used rodent models of ELS based on parental absence and disruption of maternal behavior through environmental modulation of resources (i.e., resource scarcity), respectively. Consistent with effects reported in humans, ELS alters alcohol and drug intake in developing and adult rodents [20–22]. One of the ways ELS may increase risk for SUDs is by inducing behavioral changes that promote drug use and addiction [21,23]. Indeed, there are behaviors that are linked to higher levels of drug use and augmented risk for addiction in both humans and rodents [24–35], and some of these are increased following ELS, which will be reviewed here. Moreover, many of the addiction-relevant behavioral outcomes resulting from ELS (e.g., impulsivity, drug intake, disrupted motivational processes) are also mediated by alterations within the brain’s primary reward pathway- the mesolimbic DA system. Thus, MS and LBN-induced changes in addiction-relevant behaviors and mesolimbic DA dysfunction in rodents are discussed, with a particular focus on age and sex differences given that the neurobehavioral consequences of ELS are modulated by sex (or gender in humans) and may differ based on developmental stage [36,37].

## 2. Modeling ELS in rodents

In humans, the first year of life represents a sensitive period for HPA-axis regulation and function, as cortisol activity is particularly sensitive to the quality of caregiving so that ELS involving the caregiver (e.g., neglect, abuse, maltreatment) is likely to have a large impact on subsequent brain and behavioral development [18,38]. In rodents, this period of time is thought to be analogous to the first two weeks of life, or postnatal days (PND) 1–14 [39]. The first two postnatal weeks in rodents correspond to the stress-hyporesponsive period (SHRP), during which neonates display low basal plasma concentrations of the stress hormone corticosterone (CORT) and reduced stress reactivity, as they exhibit attenuated stress-induced CORT and adrenocorticotropic hormone (ACTH) responses compared to older animals [40–43]. However, infant rats can mount elevated stress responses, as indexed by HPA-axis activation and glucocorticoid secretion, in a stressor-specific manner during this period [44,45]. MS and rearing with an LBN dam are two ELS paradigms involving the caregiver that are known to override the SHRP and compromise development across multiple levels including brain, behavioral and endocrine systems via dysregulation of the HPA-axis and other mechanisms [46–50]. This review focuses on ELS in the form of MS and/or LBN occurring during the first two weeks of postnatal life in rodents, as it constitutes a critical period for programming neurobehavioral development [46,47,49], including addiction-relevant behavioral outcomes and reward-related circuitry [21,51–53]. Below is a brief overview of the MS and LBN models (for reviews, see [47,50,54,55]).

### 2.1. Maternal separation (MS)

In this manuscript, the term MS will refer to lengthy repeated separations of neonatal rodents from their mother during the first 2–3 weeks of life. This distinction is made given that MS can occur during an acute form consisting of a single 24-h separation within the first 2 postnatal weeks, in repeated brief (3–15 min) episodes, which have been referred to as handling, or longer (6–8hr) periods per day. Importantly, the effects of MS often differ based on the length of separation but also on the pup’s developmental timing (i.e., age range) during separation [56–59]. Brief periods of MS increase the amount of pup-directed maternal behaviors (i.e., licking and grooming) when the pup is placed back in the nest [60,61], and often produce opposite hormonal and behavioral effects than those observed following extended (3–6 h) periods [57,62,63]. The burst in pup-directed maternal behavior following brief separation (i.e., handling) is thought to confer resilience to pups, as suggested by decreased stress-induced hormonal and behavioral responses as well as enhanced negative feedback of the stress response compared with non-separated pups or pups separated for longer bouts of time [56,63–69]. However, when pups are maternally deprived for extended periods of time, this results in increased HPA responsiveness and prolonged HPA-axis activity (due to the lack of a mature negative feedback system) and can result in long-term impairments in behavioral and endocrine responses to stress [56,70,71]. Here, I focus on long-term (3–4 hr) repeated MS within the first 2–3 postnatal weeks given that this manipulation often produces robust and long-lasting effects on behaviors of interest.

### 2.2. Limited bedding and nesting (LBN)

This model was originally developed by the Baram laboratory, is based on creating an impoverished cage environment via resource depletion and has been adapted by multiple laboratories worldwide [54,72]. Since bedding type and volume are critical components of the rat dam’s nesting environment, drastically reducing the amount of available bedding represents a form of chronic stress for the dam and her pups [72,73,74]. This procedure was developed to experimentally produce fragmented, adverse maternal care to mimic the quality of care that often characterizes severely stressed mothers [54]. Notably, this is a model of continuous stress exposure in pups in which the caregiver is present (unlike in the MS model) but exhibits aberrant, unpredictable maternal behaviors, including increased negative pup-directed behaviors (e.g., stepping, dragging, improper transport, shoving), thereby resulting in upregulation of the HPA-axis in pups [72–77]. This atypical infant response (e.g., increased CORT levels) is thought to contribute to immediate and later life effects of LBN on neurobehavioral function since blocking the infant increase in CORT during LBN prevents LBN-induced deficits in pup social behavior [78], although long-term effects of this manipulation (i.e., CORT blockade) remain to be determined.

The findings obtained using these two models of ELS involving disrupted caregiving are consistent with the notion that maternal presence and caregiver sensitivity are critical for regulating HPA-axis activity and maintaining low CORT levels in infant rodents, but also for determining offspring physiological and behavioral responses to stressors throughout the lifetime [79,80]. Moreover, these models recapitulate two factors thought to contribute to long-term effects of ELS on brain and behavior: 1) the deprivation of stimuli critical for behavioral and neurobiological development and 2) the effects of chronic stress exposure during a sensitive period [16,46,62], both of which may contribute to increased risk for numerous detrimental health outcomes, including SUDs and addiction. Finally, both MS and LBN are known to induce enduring changes in HPA-axis function, including later life responses to stress and drugs of abuse [47–49,71,81]. This is significant given that effects of ELS on addiction-relevant and reward-related behaviors as well as dopaminergic circuits in humans are thought to be partially mediated by alterations in HPA-axis function [7,15,16]. Thus, the neurobiological sequelae of ELS in MS and LBN rodents appears to parallel what is observed in humans and may provide opportunities to gain unique behavioral and neurobiological mechanistic insights, which may potentially aid in developing interventions to reduce risk for SUDs.

## 3. Effects of ELS on addiction-relevant behavioral outcomes in rodents

In humans and rodents, there are behavioral traits that are strongly associated with, and even predictive of, increased susceptibility to drug use and addiction [24–35]. In rodents, behavioral phenotypes associated with addiction-like behavior include increased anxiety-like behavior, impulsivity, novelty-seeking and risk-taking, sensitivity to locomotor activating properties of psychostimulant drugs, changes in alcohol and drug intake patterns as well as alterations in responses towards natural rewards (i.e. anhedonia) [24,25,52,82–88]. Importantly, the expression of behavioral traits associated with addiction-like behaviors often varies between the sexes and across development, which may provide additional insights into the links between ELS and increased susceptibility to SUDs. For these reasons, addiction-relevant behavioral outcomes resulting from ELS in the rodent models described above (i.e., MS, LBN) are reviewed, reported age and sex differences are emphasized and opportunities for future research are highlighted.

### 3.1. Effects of ELS on anxiety-related behaviors

Increased anxiety is a risk factor for developing SUDs in humans and addiction-related behaviors in rodents [25,83,85,89,90]. In humans, clinical and epidemiological research demonstrate a strong association between anxiety disorders and susceptibility for SUDs during adolescence and adulthood [91–93]. For example, anxiety disorders often precede SUDs and are also comorbid (co-occur) with SUDs [89,92,94–96]. Furthermore, ELS is a potent risk factor for the onset and persistence of both anxiety-related disorders and SUDs during later life [9,97–100]. In rodents, ELS frequently results in long-term changes in anxiety-related behaviors that can be measured using a variety of behavioral assays. These are described and summarized below.

Many rodent studies assessing ELS effects on anxiety-like behavior have employed the elevated plus maze (EPM). In this test, reductions of time spent in the open arm and in the number of open arm entries are considered indices of anxiety-like behavior [101,102]. MS decreases open arm time and open arm entries in periadolescent and adolescent male and female rats [103–106], although results showing no difference in MS groups [104], or even increased open arm time in MS females [107], have also been reported. In adulthood, both male and female MS rats were less likely to explore open arms, as they made fewer entries into the open arm and spent less time on the open arm [108–114]. Studies assessing sex differences in adult MS rats have found either decreased open arm time and entries only in MS males or decreased open arm entries and time in both males and females compared their non-MS counterparts, but also greater decreases in MS males compared to MS females [115,16]. A study focused on adult female rats found comparable open arm time and open arm entries in MS females and non-separated females [117]. In mice, studies have shown no change in MS males or females during adolescence [118], but decreased percentages of open arm time in adult MS male and female mice [119], as well as sex-specific effects during adulthood in which females show reduced open arm duration and number of entries [120]. Taken together, these data suggest that MS consistently increases anxiety-like behavior in the EPM, with male rats showing more robust effects compared with females; and that this effect may be reversed in mice, with females being more susceptible to MS effects.

Other tests used to measure anxiety-like behaviors in rodents include the light-dark box and the open field. These tests are based on rodent aversion to brightly illuminated and exposed places, which constitute a risk for predation [121–123]. In the light dark box, longer latencies to enter the light chamber and less time spent in the brightly light box are considered reflective of anxiety-like behavior. In the open field, increased time in the periphery (corners) and reduced time in the center are interpreted as increased anxiety-like behavior. In the light-dark box, prepubertal (PND 30–31) MS rats (group including both sexes) exhibited shorter latencies to enter the dark chamber and increased time spent in the dark chamber, but no changes in time spent in the center of an open field [124]. During adolescence (PND 42), only MS male rats exhibited reductions in the number of entries and time spent in the light box as well as fewer entries into the center zone of an open field [125], suggesting enhanced anxiety-like behavior only in males at this developmental stage. Decreased time spent in the light chamber has also been reported in adult MS male rats and mice compared to nonseparated or briefly separated control males [113,126–128]. However, MS does not appear to have an impact on the time spent in the light compartment or the latency to enter the light in ovariectomized adult female mice [129]. This may suggest a sex-specific effect of MS in the light dark box in male rodents across development. In the open field, many studies have reported decreased center activity (e.g., ambulation, number of visits to center, center time, latency to center, center crossings) in adult MS male rats and mice exhibited compared with non-MS males [119,127,128,130–135]. Yet, a study conducted in adult female rats found no effect of MS for time spent in the center [136], which may suggest that adult males are more sensitive to MS effects in the open field test. In mice, adult MS females exhibited reduced time spent in center [129] and number of entries into the center zone, but also greater reductions compared to MS males [119], which may suggest species-dependent effects of MS on the open field. Moreover, MS effects on adult female mice in the open field appear to be modulated by estrous cycle: MS females actually exhibited more time in the center during diestrus, but no changes compared to nonseparated females during behavioral estrus [132].

Anxiety-related behavior in rodents can also be measured using tests based on novelty-induced changes in food or water consumption in which the inhibition of food/water intake by exposure to novelty is interpreted as an index of anxiety-like behavior [137–140]. For example, MS increased novelty-induced suppression of feeding in juvenile (PND 26–29) MS rats of both sexes [141], as indexed by rats taking longer to begin feeding, and enhanced novelty-induced suppression of drinking in adult rats of both sexes [142]. MS also increased latency to feed in adult male mice [135]. Collectively, findings from MS studies in rodents suggest sex-specific effects of MS on anxiety-like behavior that differ based on the behavioral assay used: i) MS increases anxiety-like behavior in the EPM and light dark box test primarily in male rodents, although effects in rats of both sexes can be seen during adolescence; ii) MS increases anxiety-like behaviors in the open field in male rats across development (i.e., adolescence, adulthood), but in adult mice these changes are more pronounced in females and vary according to estrous cycle; and, iii) MS enhances novelty-induced suppression of feeding and drinking in rats of both sexes throughout development and in adult male mice, although effects on females remain unknown. Notably, few studies have incorporated sex as a biological variable (SABV) into their experimental design and compared the effects of MS across development and/or in both sexes within same study, underscoring the need for this type of approach.

Long-term changes in anxiety-like behaviors have also been found following LBN (i.e., scarcity-adversity), although effects are usually task and sex-specific. In the EPM, published studies have found mixed effects: no effect of LBN in adolescent male rats [143], no effect on adult male and female rats [144] or reduced percentage of open arm time or entries only in adult LBN males [145–147]. Studies conducted in adult male mice or in mice of both sexes have found no impact of LBN on the EPM [148–150]. In the light-dark box, adolescent LBN female mice exhibited reduced time spent in the light chamber, and this effect persisted into adulthood, whereas no changes were observed in adolescent or adult LBN males [126]. However, a study that used only adult male mice found reduced time spent in the brightly lit compartment of the light-dark box [149]. In the open field, there have been mixed results: no alterations in either sex during prepuberty and adulthood or a sex-specific effect in adult LBN male rats, which exhibited more time spent in the periphery and reduced time spent in the center [144,145,151]. These effects may depend on developmental timing and rodent species, as LBN exposure did not impact duration of time spent in the center in adolescent or adult male mice [143,149,150]. In mice, a study that pooled both sexes found no effect of LBN on time in center in the open field or the light dark box [152]. For novelty-induced suppression of feeding, a study that used only adult female rats found that LBN increased latencies to begin food consumption [153], whereas a study that used both sexes found no effect [151]. In mice, LBN increased latency to consume food in a novel environment only in females [150]. Taken together, these data suggest that LBN: i) increases anxiety-like behavior in the EPM and open field primarily in adult male rats, but not in mice; ii) increases anxiety-like behaviors in the light dark box that may be sex-specific and age-dependent in mice (i.e., adolescent females, adult males), although effects are relatively unexplored in rats; and iii) enhances novelty-induced suppression of feeding in adult female rodents.

In sum, ELS in the form of MS or LBN exerts long-lasting changes in anxiety-like behaviors that are typically more robust in male rodents, which exhibit increased anxiety-like behavior. It is worth mentioning that a possible contributing factor to this sex difference is that most studies have used the EPM and open field, and in female rats these tests are more reflective of activity rather than anxiety-like behavior since they are more active [154–156]. For instance, females display greater distance moved and velocity in the open field test and greater baseline velocity of movement and a higher number of open arm entries in the EPM [155,157]. Thus, caution should be exercised when interpreting results from female rats in these tests (i.e., EPM, open field). MS and LBN converged in enhancing novelty-induced suppression of feeding/drinking in both sexes (MS) or specifically in female rodents (LBN). Most studies have focused in adolescent and adult male animals or used groups pooling both sexes. Given the known sex differences in anxiety-like behavior across development and in multiple behavioral tasks, which often result in distinct and/or opposite effects, these data highlight a need for additional studies integrating SABV as a factor as well as earlier (and multiple) timepoints. Furthermore, studies assessing the relationship between ELS, anxiety-like behavior and drug intake are scarce, and may represent an opportunity to evaluate the relationships between these variables.

### 3.2. Effects of ELS on impulsivity

Impulsivity is typically characterized by rapid decision-making, premature actions, behaving without forethought, and preference for immediate over delayed gratification [158,159]. Impulsivity is commonly observed in drug users and SUDs [160–165], and has been linked to earlier emergence of and higher rates of substance abuse as well as greater substance-related adverse consequences [159,166–169]. Importantly, developmental changes in impulsive behavior are thought to contribute to SUDs; and impulsivity is particularly salient during adolescence in both humans and rodents [170–174]. Increased impulsivity is strongly associated with illicit substance use in young adults [175–178] and is a common outcome of ELS in humans that may increase susceptibility to SUDs [179–183]. Indeed, children who have experienced severe stressors such as physical abuse, neglect, and institutionalization show impaired impulse control as well as long-lasting changes in impulsivity-related behaviors [182–188]. For example, a recent study found significant associations between ELS and impulsive choice by using monetary delay discounting, in which ELS individuals exhibited a preference for smaller rewards sooner (i.e., greater impulsive choice) [185]. Collectively, these data suggest that ELS may confer risk for substance use and SUDs in humans due to effects on impulsivity.

Links between impulsivity, drug self-administration and drug-seeking behaviors have also been found in animal models useful for the study of addiction [189–192] and in rodent models of ELS [193]. Commonly used behavioral measures of impulsivity in rodents often measure impulsive action, which refers to the diminished ability to withhold a motor response, and impulsive choice, which is reflected in a preference for smaller faster rewards over larger delayed rewards [24]. Impulsive action is measured by using inhibitory control tasks such as go-no-go and stop-signal reaction time [24,189]. In these tasks, subjects are required to inhibit motor responses when a cue or stop signal is presented; and subjects who have difficulty inhibiting their response are said to display greater impulsive action [24, 89]. Impulsive choice is frequently measured by using delay-discounting tasks in which subjects must choose between smaller immediate rewards and larger delayed rewards [190]. In rats, ELS in the form of total maternal deprivation and isolation (with minimal simulated licking) results in the inability to withhold premature responses during a differential reinforcement of low rates of responding (DRL) task in adult rats of both sexes [194]. On a DRL schedule, rats are rewarded for making operant responses after a specific (minimum) length of time has elapsed. Premature responses reset waiting times and do not produce reward. The premature responses observed in adult male and female rats exposed to ELS included lower efficiency ratios for earning rewards (i.e., food pellets), shorter interresponse times, and greater number of responses during the waiting period. All of these are thought to reflect impulsive action as rats failed to inhibit their behavioral responses. However, ELS effects on impulsive choice were found to be sex- and task-dependent as only male rats who underwent maternal deprivation and minimal stimulated licking exhibited changes in delay discounting, which reflects impulsive choice [194]. Specifically, male rats who underwent maternal deprivation and minimal simulated licking exhibited higher preference for the larger reward, suggesting reduced impulsive choice [194]. Thus, this modified MS paradigm enhanced impulsive action in both sexes but reduced impulsive choice only in males. Reductions in impulsive choice, as indexed by preference for the larger delayed reward, have also been found in adult LBN male but not LBN female rats [193] or in adult LBN female rats [195]. The discrepancy observed in female rats may be due differences in prenatal stress exposure (dams bred in house vs shipped while pregnant) or the type of LBN paradigm used (continuous with the same dam vs 1-hour daily with a different dam). Unlike the modified MS paradigm, LBN had no effect on impulsive action, as measured by premature responding in the 5-choice serial reaction time task (5CSRTT), in adult male or female rats [195]. 

In sum, MS appears to increase impulsive action in both sexes but reduces impulsive choice only in male rodents whereas LBN does not appear to affect impulsive action but may reduce impulsive choice in both sexes. More studies are needed to confirm these findings and determine the ontogeny of impulsivity-related behaviors under normative conditions and following ELS to evaluate if effects are similar throughout the lifespan in rodents. For example, whether impulsivity measures are potentiated in adolescent animals following ELS is unknown. Interestingly, increased impulsivity is predictive of compulsive drug taking in rodents, although effects depend on impulsivity type (impulsive action vs choice), drug class and even sex [190]. Thus, impulsivity may be a mediating factor between ELS and drug use vulnerability in animal models relevant to addiction, but more studies are needed. Examining this possibility requires additional studies aimed at dissecting the relationships between these variables (e.g., impulsivity type, sex, drug) in ELS animals across development.

### 3.3. Effects of ELS on novelty-seeking and risk-taking behaviors

Novelty-seeking (also called sensation-seeking) is a behavioral construct related to impulsivity that refers to exploratory behavior in search of varied, novel and rewarding sensations, experiences and/or stimuli [196,197]. In humans, sensation-seeking increases vulnerability to SUDs and novelty-seeking scores are elevated in individuals with SUDs [198–200]. Moreover, men exhibit higher sensation-seeking behaviors than women during both adolescence and adulthood, suggesting sex differences in novelty-seeking behaviors that persist throughout the lifespan [201,202]. In rats, high novelty reactivity and preference is linked to individual vulnerability for developing compulsive psychostimulant administration [84,203–205]. Importantly, humans and rodents also exhibit developmental changes in novelty-seeking and risk-taking behaviors, and these behaviors are particularly salient during adolescence [201,206–213]. Increased exploratory drive and heightened novelty-seeking may contribute to increased risk-taking behavior, including drug use, and augmented addiction vulnerability during this period [214,215]. For instance, illicit drug use in humans is more common in high novelty-seeking adolescents, and adolescents exhibit increased reckless/risky behaviors compared to adults [207,216,217].

In rodents, novelty-seeking usually refers to the intrinsic drive to explore complex or novel stimuli and areas [218–220]. Novelty-seeking and preference in rodents has been compared to sensation-seeking in humans and is strongly correlated with drug self-administration and other addiction-relevant behaviors such as impulsivity and drug intake. For example, individual differences in rat behavioral responses to novelty (i.e., locomotion, preference) can predict subsequent behavioral and dopaminergic responses to cocaine as well as amphetamine self-administration in both male and female rats [88,221–224]. These studies have established a link between high novelty preference and greater psychostimulant-induced behavioral reactivity and self-administration. Yet, little is known regarding how ELS may affect the link between novelty-seeking and drug addiction in humans or rodents.

Novelty-seeking and risk-taking behaviors can be evaluated in rodents by assessing preference for novel vs familiar environments using activity chambers that differ in visual cues and/or by measuring locomotor activity in response to a novel environment [25,204]. The Concentric Square Field (CSF) consists of several arenas that stimulate exploration and risk-taking behaviors in a novel apparatus. In this test, the animal is placed in an open arena that includes sheltered and open areas, ground and elevated areas, enriched areas as well as differently illuminated zones; and, its choice for different environments is measured within the same session [225]. Elevated and/or exposed areas (e.g., bridge, center circle) are considered risky compared to dark corners since rodents tend to avoid open, elevated areas such as in the EPM [121,122]. Thus, measures associated with these areas such as number and duration of visits are a considered as an index of risk-taking behaviors [225]. During the CSF, MS increased exploratory and risk-taking behaviors in adult male rats, as indexed by shorter latencies for initial zone visits, including risky zones, and longer duration of risky zone visits compared to non-separated or briefly separated rats [226]. This is consistent with a study showing increased exploratory behaviors (i.e., nose poking, locomotion) in adult male MS rats in response to a novel environment [227]. Adult female rats exposed to long or short MS exhibited comparable behavior in the CSF, suggesting MS exerts sex-dependent effects on the CSF in which males show enhanced novelty and risk-taking behaviors [228]. However, adult female rats exhibited heightened exploratory activity specifically during high estrogen phases (i.e., proestrus, estrus) compared to diestrus [228], suggesting that fluctuations in ovarian hormones modulate the expression of exploratory and risk-taking behaviors in the CSF. With regards to sex differences in exploratory drive in response to a novel environment, adult MS females showed increased spontaneous ambulation (i.e., horizontal activity) compared to nonseparated females, whereas no effect was found in adult MS males compared with nonseparated males, as well as elevated rearing (i.e., vertical activity) compared to MS males [229]. Little is known regarding the impact of MS on the CSF at earlier developmental stages.

Other behavioral measures of novelty-seeking include the playground maze (PGM) and novel object retrieval tasks under risky conditions. The PGM is a variation of the novel object recognition test. In the PGM, exploratory responses to a single novel object are measured within the context of responses to 7 familiar objects in a familiar environment (open arena). In the initial trials, the rat is placed in the maze containing 8 novel objects (typically plastic toys) that the rat can explore/interact with. On test day, one of the familiar objects is replaced with a novel object and the time spent around the novel object area is expressed as a percentage to determine novelty-seeking responses [88,230]. A baseline sex difference has been observed in control males compared to control females during adolescence, which is characterized by a higher percentage of time spent interacting with the novel object [105]. However, only adolescent female MS rats exhibited enhanced novelty-seeking responses, as indexed by higher percentages of novel object exploration/interaction time and greater number of approach responses to the novel object, compared to control adolescent females [105]. In a novel object retrieval task in which rats had to cross an unsafe, risky zone (filled with water) to be able to access, interact with and retrieve hidden novel objects, adolescent MS rats (pooled group including both sexes) exhibited reduced latencies to cross the risky zone and reach the platform containing novel objects, took less time to retrieve novel objects, and retrieved more objects than controls [124]. A prior study conducted in adult LBN rats of both sexes revealed that LBN males spent more time exploring a novel object compared to control males, but no differences were observed in LBN females compared with control females [195]. Furthermore, although rats typically show a preference for novel environments over familiar ones [231], LBN decreased preference for a novel environment in adult rats of both sexes [195]. These findings suggest that LBN increases novelty-seeking behavior in response to novel objects in adult male rodents but reduces preference for novel environments in both sexes, although more studies are needed to confirm these findings.

With regards to the relationship between ELS and novelty-seeking in rodents, results obtained using the MS model suggest that ELS produces sex-specific increases that are task- and developmentally dependent. Adult males exhibited increased novelty-seeking and risk-taking behaviors during the CSF. However, MS effects on exploratory behaviors in response to a novel environment were augmented in female MS rodents compared to male MS rodents and varied according to estrogen levels. In the PGM, adolescent MS females showed an enhancement in novelty-seeking behaviors. In novel object retrieval tasks, adolescent MS animals exhibited enhanced novelty-seeking and risk-taking behaviors, but sex differences remain unexplored. LBN decreased preference for novel environments in both sexes, but a sex-dependent effect was found in response to novel objects in which male LBN rodents exhibited increased exploration.

### 3.4. Effects of ELS on alcohol intake

Early life environments are known to modulate later life patterns of alcohol consumption in both humans and rodents. Humans with a history of ELS, including parental absence, maltreatment, and neglect, are more likely to start drinking early in life and use drinking as a coping mechanism [13,14,232–234]. This is significant given that earlier initiation of alcohol consumption is correlated with increased susceptibility to alcohol dependence and abuse in both males and females [235–237]. A study examining sex differences found that ACEs were associated with excessive alcohol use, but sex did not moderate this relationship, suggesting that ACEs impact alcohol use similarly in both sexes [238]. Thus, ELS is a potent risk factor for lifetime alcohol dependence in both men and women [13,239].

ELS also potentiates voluntary alcohol intake in rodents, and many studies have reported sex differences. MS does not appear to impact long-term voluntary alcohol intake in adult female rats [228,240]. However, adult MS male rats and mice consume more of an ethanol solution during adulthood and exhibit increased preference for alcohol over water [111,241–245]. Increased voluntary alcohol intake is also observed in adolescent MS male rats and mice compared with nonseparated controls [246,247]. Studies pooling both sexes have found that MS increases alcohol intake during an operant task in adult rats [248–250]. Interestingly, a study that identified increased anxiety-like behavior, HPA-axis hyperresponsiveness and greater alcohol consumption in adult male MS mice found that all of these measures were reduced following antidepressant administration, suggesting a link between HPA-axis dysregulation, negative affect states and greater alcohol intake following ELS [111]. Similar sex-dependent effects of ELS on alcohol consumption have been observed in adult LBN mice. Although adult male LBN and control mice consumed similar baseline amounts of alcohol, male LBN mice exhibited accelerated alcohol intake escalation compared with controls, but this effect was not observed in adult LBN females [251]. These data suggest that LBN exposure accelerates the transition from moderate to excessive alcohol drinking in adult males but not females [251]. In sum, MS in rodents increases adult alcohol consumption whereas LBN accelerates the transition to excessive alcohol consumption. These effects are often more potent in males and typically not observed in females, suggesting that female rodents may be more resilient to the effects of ELS on later life alcohol intake.

### 3.5. Effects of ELS on behavioral responses to drugs of abuse and drug intake

In humans, exposure to ELS increases susceptibility for early initiation of drug use as well as later life addiction [6,7]. Childhood stressors, as well as the number/frequency of childhood stressors, are strongly related to early adolescent drug use and later life drug-related problems [8–12,252]. This is significant given that adolescence is a critical period in development during which drug exposure increases risk for SUDs [171,253], and ELS is thought to increase risk for SUDs through its influence on adolescence substance use [17,254]. Thus, adolescent drug use may represent an intermediate step in the path from ELS to SUDs.

ELS in rodents alters behavioral responses to a wide variety of drugs, often in an age- and sex-dependent manner. These behavioral responses include conditioned place preference (CPP), which refers to animals preferring a context previously paired with drug reward, drug-induced locomotion, behavioral sensitization, which refers to the augmented motor-stimulant response that occurs with repeated, intermittent administration of an addictive substance as well as drug-self administration [22,255–257]. Furthermore, the influence of ELS on drug-induced responses appear to differ based on drug class, as reviewed below.

#### 3.5.1. Psychostimulants

MS influences behavioral responses to psychostimulants during later life, although effects vary depending on multiple factors including age and sex of the animals as well as the drug and dose tested. Adolescent male MS rats exhibited levels of methamphetamine-induced place preference that were similar [258] or reduced compared to those in nonseparated controls [259]. However, adolescent MS rats exhibited augmented locomotor responses (i.e., behavioral activation) to methamphetamine, but this effect occurred at different doses for male (3.0 mg/kg) and female (1.0 mg/kg) rats [260]. Adolescent MS rats also exhibited increased cocaine-induced (10 mg/kg) place preference and locomotion, but these effects were only observed in males [105,261]. It is worth noting that adolescent control (i.e., nonhandled) female animals exhibited higher levels of cocaine-induced locomotion compared to males, suggesting a sex difference at baseline (no stress) conditions [105]. Collectively, these data suggest that MS increases psychostimulant-induced locomotion particularly in males. These effects may be explained by a sex-specific developmental increase in psychostimulant-induced locomotion in females, which may contribute to a ceiling effect in these animals.

MS does not appear to impact cocaine-induced behavioral sensitization in periadolescent (PND 36–37) male rats [262]. However, adult MS rats of both sexes exhibited behavioral sensitization to amphetamine, as animals responded to the amphetamine challenge injection with increased locomotor activity, as well enhanced cocaine-induced locomotor activity (5–10 mg/kg i.p.; 1.0 mg/kg i.v.) [263,264,265]. A study conducted in adult female rats found no changes in cocaine-induced locomotion but attenuated cocaine-induced behavioral sensitization in MS females compared to nonseparated females [266]. With regards to drug self-administration, adult male MS rats exhibited increases in methamphetamine self-administration (e.g., more active lever presses, higher number of infusions) compared to adult males that experienced shorter (15-min) separations [267,268]. Effects on adult females remain to be determined. Adult MS rats of both sexes acquired cocaine-self administration at lower doses (0.0625–0.125 mg/kg) than nonseparated controls [264], suggesting that MS facilitates the acquisition of cocaine self-administration during adulthood. However, the total voluntary cocaine intake (number of infusions per session) did not differ between MS and nonseparated animals at these doses [264]. When tested at higher doses (0.125–0.5 mg/kg), adult MS rats actually exhibited reduced voluntary cocaine intake, as indexed by fewer numbers of cocaine infusions as well as blunted drug-induced reinstatement of the cocaine response, as demonstrated by MS rats making fewer presses on the formerly active lever compared to non-separated animals [269]. These data suggest that MS facilitates the acquisition of psychostimulant administration, but not total intake, during adulthood and that these effects may be limited to lower doses.

LBN also exerts long-term effects on behavioral responses to psychostimulants, but developmental and sex differences remain to be explored. Adult male LBN rats did not exhibit changes in cue or drug-induced reinstatement of cocaine-seeking, nor cocaine-induced locomotion (10 mg/kg) but acquired cocaine self-administration faster and preferred to self-administer lower doses of cocaine compared to controls under low effort conditions [270]. This is consistent with findings obtained in adult MS rats [264]. Adult male LBN mice exhibited increased locomotor activity in response to acute cocaine administration compared with controls, but comparable behavioral sensitization [271].

#### 3.5.2. Opioids

MS exposure produces robust behavioral effects in response to opioid administration. Adult male MS rats exhibited morphine-induced CPP- even to lower doses, greater morphine-induced locomotion as well as increased intake and preference of a morphine solution compared to nonseparated control rats [272–278]. With regards to morphine-induced locomotion, MS produced behavioral sensitization in males, whereas females exhibited tolerance to morphine-induced locomotor activity [276]. Adult MS female rats exhibited enhanced CPP to morphine, although this was reduced when compared to MS males suggesting a sex difference, as well as increased morphine intake compared to nonseparated control females [275–277]. Thus, MS increases opioid self-administration in males and increases behavioral responses to opioids in both sexes, but MS females appear less affected in subset of these effects (i.e., behavioral sensitization, place preference) compared to MS males.

Sex-dependent changes in opioid administration have also been reported in adult LBN rats. Although total heroin self-administration (0.1 mg/kg/infusion) during acquisition was comparable in adult female control and LBN rats, LBN exposure increased resistance to extinction as indexed by LBN female rats taking longer to reach extinction criteria [279]. Moreover, adult LBN females exhibited enhanced cue-induced and heroin-primed reinstatement of heroin-seeking behavior (i.e., more drug lever presses) [279]. In adult male rats, LBN led to reduced heroin consumption during the training phase as well as overall consumption, as indexed by lower numbers of infusions [280]. Despite reduced intake, LBN did not alter extinction or reinstatement to heroin seeking in adult males [280]. Thus, LBN potentiates heroin-seeking behaviors in female but not male rats. A study that pooled both sexes found no differences between adult LBN and control animals for acquisition and maintenance of heroin-self administration (0.06 mg/kg/infusion), but increased breakpoints for heroin self-administration under a progressive ratio reinforcement schedule, impaired extinction of heroin-seeking behavior and increased reinstatement of heroin-seeking by heroin-associated cues in LBN rats [281]. Given prior findings in adult LBN females and males [279,280], it seems likely that the effects reported in the latter study, which pooled both sexes, were driven by female-specific changes. With regards to morphine, adult LBN rats exhibit sex-specific effects in which males are resistant to opioid-seeking behavior. LBN males earned fewer infusions of low-dose morphine (0.25 mg/kg) compared to control males under both fixed and progressive reinforcement schedules, whereas LBN and control females earned similar number of infusions [193]. These behavioral effects were specific to a lower dose of morphine, as LBN did not impact the number of infusions obtained by animals of both sexes at higher doses (0.75 mg/kg) [282]. Taken together, these findings indicate that LBN increases heroin-seeking behaviors in females but reduces morphine self-administration in males, and that effects are specific to lower doses of opioids.

### 3.6. Effects of ELS on responses to natural rewards

In rodents, sweet foods/solutions and social interactions constitute natural rewards, and as such, they have potent motivational properties and activate brain’s primary reward pathway- the mesolimbic DA system [283–291]. A growing body of evidence indicates that ELS in the form of MS and LBN disrupts later life responses to rewards, including natural rewards, as measured on a variety of behavioral tasks [52,292].For instance, both male and female rats reared under ELS conditions exhibit alterations across a spectrum of reward-related processes throughout development, including intake of palatable solutions and/or foods as well as motivated social behaviors known to be rewarding to rats, including play and sexual behaviors [52]. This is in accordance with human studies showing disrupted reward-related responses following ELS [293,294,295].

#### 3.6.1. Sucrose/palatable food intake

Since developing and adult rats typically exhibit an unlearned preference for sucrose solutions over water [296,297], a way to measure changes in motivational and reward-related behaviors involves administration of sugar-containing liquids and foods. MS typically decreases intake and/or preference of sucrose solutions (1–2%) compared to control (non-separated) rats in adolescent male and adult rats [111,133,134,272,298–304], but has no effect in adolescent females or adult females [298,303–306]. However, effects depend on the concentration of the sucrose solution used, as adult MS male rats exhibited increased intake of a 3% or 10% sucrose solution compared with non-separated controls [307,308]. This suggests that ELS may reduce sucrose consumption to lower concentrations of sucrose but increases sucrose consumption at higher doses in male rodents during free choice paradigms. Adult male MS rats also exhibited reduced sucrose self-administration, as indexed by reduced lever presses, suggesting attenuated motivational drive for sucrose reward in these animals [309]. With regards to palatable food intake, studies have found reduced cookie intake in periadolescent (PND 28–40) male rats [310], but sex-specific effects during late adolescence/early adulthood: no differences between MS and nonseparated males, but MS females consumed more of a graham wafer compared to nonseparated females [107]. In adulthood, male and female MS rats exhibited increased palatable food consumption [116,311]. These findings suggest that MS induces distinct effects on sucrose vs palatable food consumption: a sex-specific decrease in sucrose consumption and preference in adolescent and adult male MS rodents, whereas it increases palatable food consumption in both sexes during adulthood.

LBN exposure within the first two postnatal weeks reduced adolescent and adult sucrose consumption and/or preference in male rats [143,312–314]. However, LBN did not alter sucrose consumption in adult female rats, as they were comparable to controls [279]. No effect of LBN was observed for sucrose preference in either sex in prepubertal (PND 30) rats [151], which may suggest that LBN-induced alterations in sucrose intake emerge later in development (~PND 45) specifically in males. The effects of LBN on sucrose intake and preference patterns are similar to those observed following MS, in which reductions are seen specifically in adolescent and adult males. In mice, LBN reduced sucrose consumption in adult females [150,315], but not males [315,316], which may suggest that LBN effects on sucrose consumption is species-dependent and that sex-dependent effects on mice are opposite from those observed in the rat. LBN also induced sex-specific effects with regards to consumption of palatable food. LBN reduced palatable food intake at baseline (no effort condition) in adult male rats [270], but not in female rats [279]. Adult LBN female rats consumed more palatable food, exhibited increased preference for palatable food, and were willing to expend more effort for palatable food pellets compared to control females [153,279]. With regards to palatable foods, LBN results in bidirectional effects in the sexes: decrease in males, increase in females.

In sum, both MS and LBN reduce sucrose preference in adolescent and adult male rats, with no effect on females at these timepoints. MS increases palatable food consumption in both sexes during later life, whereas LBN exerts sex-specific bidirectional effects: it decreases palatable food consumption in males but increases palatable food intake in females. Collectively, these findings demonstrate long-term effects of ELS on behavioral responses to natural rewards such as sucrose and palatable food.

#### 3.6.2. Social behaviors

Social interactions constitute a form of natural reward for rodents and increase activity within the same brain pathway activated by drugs of abuse- the mesolimbic DA system [317,318]. Although rodent social behaviors are diverse (see Table 1 for descriptive summary of these behaviors), here I focus on social play, social approach, social interaction and sexual behaviors given the rewarding value of these interactions and their mediation by mesolimbic dopaminergic neurotransmission [289,291]. MS exerts sex-dependent effects on play behaviors in rats, and results differ depending on the control group used. Periadolescent (PND 30–40) MS male rats have been reported to make both more and fewer nape attacks (i.e., pouncing), which are considered as play solicitations, compared to non-separated sex-matched controls [319,320]. These mixed results may be due to differences in methodology including duration of MS (2 vs 3-week), number of behavioral tests done on the same animal, testing location (home cage vs play box arena) and familiarity of the play partner (cage mate vs novel rat). Nape attacks were not affected in MS females, but MS females did exhibit reductions in pinning behavior compared to control females [319]. A study that compared MS, handled and nonhandled control rats found no effect of MS on nape attacks in juvenile (PND 24–26) male and female rats [321]. However, adolescent MS animals exhibited increased pinning behavior compared to briefly separated (15-min) male rats, whereas MS and briefly separated females made comparable numbers of pins [322]. These results suggest more robust effects on MS on pinning behavior compared to other indices of social play as well as a reduction in pinning in MS female rats compared to controls but an increase in pinning in MS males. With regards to social interaction, MS exerts sex-dependent effects in juvenile (PND 25) rats: no effect in MS males compared to nonseparated males, but MS females exhibited reduced nose-to-nose duration and increased the latency for nose-to-nose contact [323,324]. In adolescent (PND 42–45) rats, increased latency for nose-to-nose contact was observed only in MS males [323]. However, periadolescent male MS rats exhibited increased frequency of anogenital sniffing [325], suggesting MS may exert differential effects on nose-to-nose vs anogenital sniffing. In adolescent male mice, no effect of MS on affiliative behaviors has been reported [326]. Finally, although MS accelerated sexual maturation in males, the frequencies of sexual behaviors did not differ in adult MS males or females compared to nonseparated controls [327].

LBN rats exhibit alterations in social behaviors such as social play, approach (i.e., motivation), interaction and sexual motivation. For example, early life rearing with an LBN dam decreases reward-related play behaviors in adolescent and adult male rats [143,312] but not in prepubertal rats of either sex [328]. LBN rats also exhibit blunted social motivation, as indexed by reduced time spent in a chamber containing a social stimulus animal, prior to weaning as well as during adolescence and adulthood [313,329–332]. In the 3 chambered social approach test, these effects have been observed across development in LBN rats (i.e., group pooling both sexes) [331]. Adult male mice exposed to LBN also exhibit reductions in social approach behavior and time spent with a social stimulus [316]. In adulthood, sex differences have been reported for social interaction in an open arena, in which social contact time is reduced only in LBN males [144]. With regards to sexual motivation, adult LBN male and female rats spent more time with a sexually receptive female or sexually vigorous male, respectively, compared to controls during the no-contact phase of a partner preference test in which they had to choose between a same sex vs opposite sex partner [333]. Moreover, adult LBN male rats exhibited altered copulatory behaviors including reduced latencies to mount, intromit, and ejaculate at earlier timepoints than controls over a 3-week period [334], although the frequency of these behaviors did not differ between adult LBN and control males [333]. In adult female mice, LBN delayed sexual maturation [335].

To summarize, MS and LBN appear to influence different types of social behaviors. MS reduced play behaviors in females but increased them in males, whereas LBN reduced play behaviors in male rats. MS impaired social interaction at earlier timepoints in females (i.e., juvenile) than males (i.e., adolescence), but no effect was seen in mice. LBN reduced social approach and interaction across multiple time points in males and groups pooling both sexes, although effects may be driven by males considering that only LBN males exhibited reduced social interaction during adulthood. Finally, MS and LBN both accelerated sexual maturation specifically in males, and LBN enhanced adult sexual motivation in both sexes.

## 4. Effects of early life stress on later life mesolimbic DA system

The mesolimbic DA system consists of dopaminergic projections from the ventral tegmental area (VTA) to the nucleus accumbens (NAc), amygdala and prefrontal cortex (PFC), among others, and is fundamental to reward-related processes including the drug-induced sensation of pleasure that acts as a positive reinforcement signal [346,347]. Activation of the mesolimbic DA system, which stimulates VTA DA release and dopaminergic neurotransmission, is necessary for the rewarding properties of both natural and drug rewards [346,348,349]. Addiction is characterized by severe dysregulation of motivational circuits, which can include exaggerated incentive salience, reward deficits, and impulsivity-all of which involve mesolimbic DA system dysfunction [350,351]. As reviewed above (Section 3), ELS in the form of MS or LBN disrupts addiction-related behaviors in rodents (e.g., impulsivity, drug intake, behavioral responses to natural rewards) that are known to be mediated by the mesolimbic DA system. These findings suggest that ELS may influence later life addiction-relevant behavioral phenotypes via disruption of mesolimbic DA function.

ELS produces enduring effects in reward-related processes and mesolimbic DA function in humans [293,294,295]. In accordance, multiple studies have demonstrated a long-term impact of ELS, as induced by disrupted mother-infant interactions due to MS or LBN, on the mesolimbic DA system in rodents (for reviews, see [51,53]). For example, MS results in robust and long-lasting changes in DA transporter (DAT) levels in the NAc, which were reduced in MS rats compared to controls, as well as in the responsiveness of mesolimbic DA neurons to stress, in which MS rats responded to a mild stressor (tail pinch) with significantly greater increases of NAc DA levels [263,352]. It is worth noting that DA responses to stress are dependent on the developmental stage, duration and type of stressor used, as NAc and VTA DA responses to 1-hour restraint stress were suppressed in adolescent MS rats [353]. MS rodents also exhibit changes in DA concentrations and DA receptor expression during later life, although effects often differ by sex, age, receptor subtype and brain region [106,243,244,311,354–358]. Stereological analysis of VTA neurons in adult MS rats (group containing both sexes) have identified a reduction in tyrosine hydroxylase (TH)-positive DA neurons [250], whereas a study that used both sexes found increased number of DA neurons, as indexed by TH-immunoreactivity, in adult MS females but not males [265]. Moreover, MS also results in long-lasting alterations in the electrical activity of VTA DA neurons in both sexes. Using *in vivo* extracellular recordings, Masrouri and colleagues found that adult MS males displayed a decrease in firing rates and burst firing compared to control males, suggesting VTA DA hypoactivity [359]. Using whole-cell patch clamp recordings in brain slices from adolescent female rats, Spyrka and colleagues found an MS-induced increase in VTA DA neuron excitability and enhancement of excitatory synaptic transmission [360]. These findings may suggest opposite effects of MS on VTA DA neurons in males and females (i.e., lower numbers and hypoactivity in males, higher numbers and hyperactivity in females). Taken together, these findings demonstrate that MS alters the mesolimbic DA system across different levels in rodents (e.g., release, receptors, neurons) and that sex differences are present in these effects.

Available evidence suggests a long-term impact of LBN on later life mesolimbic DA system structure and function. For example, adolescent and adult LBN rats (group containing rats of both sexes) exhibited blunted c-Fos immunoreactivity, an indirect marker of neural activity, during a social approach test within cortical (PFC), striatal (NAc), and limbic (basolateral amygdala-BLA) structures receiving dopaminergic innervation from the VTA [331], which may suggest blunted mesolimbic DA system activation in response to social stimuli. With regards to drug-induced neural activity within reward-related structures, a recent study conducted in adult LBN males showed differential heroin-induced c-Fos expression, as indexed by blunted heroin-induced neuronal activation in the NAc core, but not the NAc shell, in LBN males compared to controls [280]. This finding is in accordance a prior study showing reduced excitatory activity in the NAc core of adult LBN males but not adult LBN females [193]. Adult LBN males also exhibit alterations in NAc DA receptor density, as measured by immunoreactivity [361]. Neuroimaging studies conducted in LBN rats have identified structural, morphological, functional connectivity changes within nodes of the mesolimbic DA system [144,312,313,362]. For instance, preweaning (PND20) LBN male rats, but not female rats, exhibit morphological changes in the BLA characterized by increased spine density, dendritic length and arborization compared to CON males as well as BLA hyperexcitability in vitro [144]. Male LBN rats also exhibit reduced resting state functional connectivity between cortical (mPFC) and limbic (BLA) structures, between the BLA and VTA, as well as between cortical (mPFC) and striatal reward-related areas (caudate putamen) compared with CON rats [313,362]. These data suggest that functional connectivity within multiple components of the mesolimbic DA system is altered by early life LBN exposure, which may contribute to impaired reward processing underlying reward-related deficits in LBN rats such as reduced sucrose consumption and blunted social play [313]. Finally, LBN also results in enduring changes in the activity of VTA DA neurons: adult LBN female rats exhibited a reduction in the number of spontaneously active VTA DA neurons (i.e., population activity) compared with control females, suggesting a hypodopaminergic state [77]. Whether these findings (i.e., DA downregulation) extend to adult LBN male rats remains to be determined. Within this context, a decrease in VTA DA neuron activity would be expected to diminish stimulus-driven DA neuron responses, leading to attenuated mesolimbic DA system activation and blunted reward-related responses [363]. Notably, hypodopaminergic states are also observed in adult male and female rats exposed to chronic stress [364,365], in which VTA DA attenuation is driven by BLA hyperexcitability, and specifically, increases within a BLA-ventral pallidum inhibitory circuit that decrease VTA DA neuron activity [364,366]. Thus, an interesting possibility is that this circuit also contributes to LBN-induced changes in VTA DA neuron function, but this remains to be tested.

In sum, results obtained using MS and LBN in rodents indicate that the maturation of the mesolimbic DA system is sensitive to ELS, as suggested by broad and long-lasting changes in structure and function within nodes of this system, including its source (i.e., VTA DA neurons). Thus, ELS interferes with mesolimbic DA system development and disrupts later life function. The mechanisms by which ELS disrupts mesolimbic DA system development and function remain unclear, although ELS-induced changes in gonadal and stress hormone signaling systems known to regulate mesolimbic DA activity have been implicated [367–369]. Other mechanisms including ELS-induced changes in transcriptomic patterns across the reward circuitry [370], glutamatergic signaling [193,371], and microglial pruning of stress-related brain regions [372,373] have also been suggested.

## 5. Conclusions

In rodents, ELS in the form of MS and LBN results in HPA-axis dysregulation and long-lasting changes in addiction-related behavioral phenotypes including anxiety-like behavior, impulsivity, novelty-seeking, as well as altered behavioral responses to alcohol, drugs and natural rewards. These findings are briefly summarized here and in Table 2. MS typically increases anxiety-like behavior in male and female rodents in a variety of tasks (e.g., EPM, open field, light-dark box, novelty-induced suppression of feeding), although males display anxiety-like behavior in more tests than females. LBN tends to increase anxiety-like behavior in both sexes, but this effect is task-dependent (EPM and open field in males, light-dark box and novelty-induced suppression of feeding in females). In terms of impulsivity, MS increases impulsive action in both sexes but reduces impulsive choice only in males; LBN spares impulsive action but reduces impulsive choice primarily in males as mixed findings have been reported for females. Within the context of novelty-seeking and risk-taking in rodents, adult MS males exhibit increased novelty-seeking and risk-taking behaviors in the CSF, although adolescent MS animals of both sexes exhibit enhanced novelty-seeking and risk-taking behaviors in novel object retrieval tasks and adolescent MS females exhibit enhanced novelty-seeking in the PGM. LBN decreased preference for novel environments in both sexes. ELS increases alcohol intake (MS) or accelerates the transition into excessive alcohol consumption (LBN) in males but not females. The impact of ELS on later life responsivity to drugs and drug intake depends on the stressor paradigm used and the animal’s sex. MS increases addiction-related behavioral responses to (i.e., CPP, drug-induced locomotion) and self-administration of psychostimulants and opioids in both sexes, although these effects are typically more robust in males. Following LBN, males exhibit reduced intake of psychostimulants and opioids as well as reduced cue and drug-induced opioid reinstatement whereas females do not exhibit changes in opioid intake but display enhanced cue and drug-induced reinstatement of opioid-seeking behaviors. ELS reduces sucrose preference in adolescent and adult male rats, with no effect on females. MS increases palatable food consumption in both sexes during later life, whereas LBN exerts sex-specific bidirectional effects: it decreases palatable food consumption in males but increases palatable food intake in females. Finally, ELS tends to decrease social play behaviors and dampen social motivation for a conspecific in both sexes. As discussed here, the effects of ELS on addiction related-behavioral outcomes are often sex-dependent and can vary across the animal’s lifespan (see Table 2 for summary of behavioral outcomes following MS and LBN in male and female rodents). For example, adult males exposed to ELS often exhibit heightened anxiety-like behavior, increased novelty-seeking and risk-taking, greater alcohol intake, and dampened responses to natural rewards, but the effects on drug responding/intake (potentiation vs reduction) depend on the ELS paradigm used and drug type. Importantly, the findings reviewed here demonstrate long-term effects of ELS in responses to natural rewards such as sucrose, palatable food and social encounters, which is in accordance with a recent meta-analysis indicating dampened reward responsiveness and social deficits following ELS in rodents [292] and clinical studies showing disrupted reward processing in adults previously exposed to adverse early life experiences [53,293,294].

In addition, ELS influences the development of the mesolimbic DA system, which undergoes protracted development and is thought to play a pivotal role in numerous addiction-related outcomes resulting from ELS. As such, it is likely that ELS increases susceptibility for later life drug use and addiction by interfering with the normal maturation of reward-related brain and behavioral function. This would suggest that mesolimbic DA function may be a target for modulating the effects of ELS on later life addiction-related behaviors including alcohol/drug intake. Yet, little is known regarding the mechanisms by which ELS disrupts mesolimbic DA system development and DA signaling. Indeed, most behavioral and neurobiological studies have focused on assessing neurobehavioral outcomes in only one sex (i.e., males) at one developmental timepoint (i.e., adulthood), so the relationship between ELS, addiction-related behaviors, drug use and DA function remains poorly understood. A comprehensive understanding of how ELS influences behavioral and DA system development in both sexes is critical for understanding the mechanisms by which ELS potentiates risk for later life SUDs.

## Figures and Tables

**Table 1:** Social Behaviors Modulated by ELS.

**Social Play:** This test consists of observing a pair of rats under baseline conditions or following a social isolation period, which is known to increase play behaviors [336]. Play behaviors can be measured in the home cage or in a neutral arena and recorded. These include following and chasing, pouncing or nape contacts (when one animal contacts the back of the neck of the other animal with its paws or snout), bouts of boxing/wrestling (also called rough and tumble play or play fighting), as well as duration and frequency of pinning (when one rat exerts a dominant posture over the other rat by holding it down with its back against the floor). Nape contacts and pins are considered the main indices of social play behaviors [337]. Nape contacts are considered index of play solicitations whereas pins are usually regarded as the culmination and possibly extension of a play bout. This test serves as an index of social reward and is useful for measuring future dominance hierarchies [318,338]. Reductions in social play are often interpreted as indicative of blunted social reward-related behavioral and brain function. **Social Approach:** This test occurs in a 3-chambered apparatus in which the test animal is placed in a center chamber that is in between two other chambers [339,340]. Both side chambers contain an enclosure that enables the test animal to see and smell its content but prevents direct contact. During testing, one enclosure contains a conspecific animal while the other one is empty or contains an inanimate object. In this test, the amount of time an animal spends exploring each enclosure (sniff time) and/or each chamber containing an enclosure (social chamber vs control chamber) is recorded. Decreases in time spent sniffing the enclosure containing the conspecific animal (or the chamber containing this enclosure) are interpreted as attenuated social motivation and/or social avoidance [341,342]. **Social Interaction:** In contrast to the social approach test, which limits the degree of social interactions possible, this test allows for the measurement of direct social interactions as the animals can freely explore each other [342,343]. Several behaviors such as sniffing, following, crawling over and under, contact time and grooming are measured and recorded. Reductions in social interaction are commonly interpreted as social withdrawal and/or avoidance [341]. **Sexual Behavior:** Sexual behaviors are typically measured in male rats by presenting them with a sexually receptive female rat- a naïve, virgin, estrus female rodent. In males, sexual behaviors include mounting (the male straddles the female from behind and thrusts his hips), intromission (mounting with vaginal insertion) and ejaculation, including the number of and or latency to reach these behaviors [344]. In females, sexually receptive measures are centered around lordosis (a receptive posture that facilitates intromission)[345]. These behaviors are usually recorded/videotaped and scored.

**Table 2:** Summary of Behavioral Findings after ELS in rodents.

Behavioral Outcome/Sex	Males	Females
Anxiety-like behavior	MS EPM: ↑ Open Field: ↑ Light-dark box: ↑ Novelty-induced suppression of feeding/drinking: ↑	MS EPM: ↑ Open Field: - rats, ↑ mice Light-dark box: - Novelty-induced suppression of feeding/drinking: ↑
	LBN EPM: ↑ adult rats, - mice, Open Field: - prepubertal rats, mice; ↑ adult rats Light-dark box: - rat, ↑ mice Novelty-induced suppression of feeding and drinking: -	LBN EPM: - Open Field: - Light-dark box: ↑ mice Novelty-induced suppression of feeding and drinking: ↑
Impulsivity	MS Impulsive choice: ↓ Impulsive action: ↑	MS Impulsive choice: - Impulsive action: ↑
	LBN Impulsive choice: ↓ Impulsive action: -	LBN Impulsive choice: - Impulsive action: -
Novelty-Seeking/Risk-Taking	MS CSF: ↑ Risky novel object retrieval: ↑	MS CSF: - Risky novel object retrieval: ↑
	LBN Novel object exploration: ↑ Preference for novel environment: ↓	LBN Novel object exploration: - Preference for novel environment: ↓
Alcohol Intake	MS Consumption: ↑ Preference: ↑	MS Consumption: - Preference: -
	LBN Intake escalation: ↑	LBN Intake escalation: -
Drug Intake and Response	MS Psychostimulant-induced CPP: ↓ methamphetamine, ↑ cocaine Psychostimulant-induced locomotion: ↑ methamphetamine, cocaine Psychostimulant self-administration: ↑ intake methamphetamine, - intake low dose cocaine, ↓ intake high dose cocaine Opioid-induced CPP: ↑ Opioid-induced locomotion: ↑ Opioid intake: ↑	MS Psychostimulant-induced CPP: - cocaine Psychostimulant-induced locomotion: ↑ methamphetamine, - cocaine Psychostimulant self-administration: - Opioid-induced CPP: ↑ Opioid-induced locomotion: - Opioid intake: ↑
	LBN Psychostimulant-induced locomotion: - rats, ↑ mice Psychostimulant self-administration: ↓ intake Cue and drug-induced reinstatement: - Opioid intake: ↓	LBN Psychostimulant-induced locomotion: ? Psychostimulant self-administration: ? Cue and drug-induced reinstatement: ↑ Opioid intake: -
Response to Natural Rewards	MS Sucrose: ↓ Palatable Food: ↓ prepubertal, - adolescents, ↑ adults Social: - juvenile play, ↓ prepubertal play, ↑ prepubertal/adolescent play; - interaction (juvenile rats, mice), ↓ adolescent interaction, - frequency of sexual behaviors	MS Sucrose: - Palatable Food: ↑ Social: - juvenile play, ↓ prepubertal play; ↓ juvenile interaction, - adolescent interaction, - frequency of sexual behaviors
	LBN Sucrose intake: ↓ rats, - mice Palatable Food: ↓ Social: ↓ play, ↓ approach, ↓ interaction, - frequency of sexual behaviors	LBN Sucrose intake: - rats, ↓ mice Palatable Food: ↑ Social: ↓ play, ↓ approach, ↓ interaction, - frequency of sexual behaviors

MS- maternal separation, LBN- limited bedding and nesting. ↓ most studies indicate a reduction, ↑ most studies indicate an increase, - indicates no change compared to control animals, ? indicates an unknown.

## Data Availability

No data was used for the research described in the article.
